# Items

**Published:** 1878-11

**Authors:** 


### Items.

—Balata, a substitute for gutta percha, which it closely resembles, has recently attained importance in commerce. It is obtained from a tree that grows upon the banks of the Orinoco and the Amazon rivers of South America, after a mann r similar to that of obtaining caoutchouc and gutta percha. The *Boston Journal of Chemistry*, in describing the characters of the substance, says, that though inferior to caoutchouc in extent of its uses, it has points of superiority over gutta percha. It gives an agreeable odor when warmed, is flexible and more elastic than gutta percha, may be joined piece to piece at a temperature of 120° Fahrenheit, but requires 270° for melting, a higher point than for gutta percha. It has various solvents, and will be valuable for insulating purposes.

—A common formula for preparing Hamburg Tea, according to a correspondent of *New Remedies*, is: Senna leaves, eight parts; Manna, four parts; Coriander, one part. Mix.

—Chlorhydrate of pilocarpine, the active principle of the powerful diaphoretic, jaborandi, is quoted at wholesale at forty cents a grain, and is obtainable in Buffalo.

—At the last session of the medical examining board of the U. S. Marine Hospital Service at Washington, in August, thirteen out of thirty-one applicants appeared, and of these, three were accepted and appointed assistant surgeons.

